# Radiological findings spectrum of asymptomatic coronavirus (COVID-19) patients

**DOI:** 10.1186/s43055-020-00266-3

**Published:** 2020-08-18

**Authors:** Rasha Mostafa Mohamed Ali, Mai Bahgat Ibrahim Ghonimy

**Affiliations:** Diagnostic & interventional Radiology Department, Thoracic & Gastrointestinal Tract Imaging units, Kasr Alaini Hospitals, Cairo University, Cairo, Egypt

**Keywords:** Coronavirus disease 2019 (COVID-19), Asymptomatic patients, Computed tomography

## Abstract

**Background:**

Radiological examinations of COVID-19 positive patients play a vital role in early diagnosis and assessment of disease course, as most COVID-19 infected patients were diagnosed with pneumonia and characteristic CT imaging patterns. Asymptomatic infected individuals, called “asymptomatic carrier or transmitter”, who are the infectious sources of SARS-CoV-2, and some of them progress rapidly, even resulting in acute respiratory distress syndrome (ARDS) with a high case-fatality rate. Our study is a prospective study and aims to be familiar with the CT imaging features of asymptomatic cases with COVID-19 pneumonia.

**Results:**

A total of 44 asymptomatic cases with COVID-19 pneumonia between March 20, 2020 and May 23, 2020 were enrolled. All patients had a history of exposure to SARS-CoV-2 or recent travel history. All patients had no symptoms. The predominant feature of CT findings in this cross-sectional study was ground-glass opacity (GGO) (41, 93%) with peripheral (34, 77.3%) distribution, bilateral location (20, 45.5%) with lower lobe predominance (left lower lobe 72% and right lower lobe 50%).

**Conclusion:**

CT imaging of asymptomatic cases with COVID-19 pneumonia has highly characteristics findings. Since asymptomatic patients are the asymptomatic transmitter, and some patients can progress rapidly in the short term, it is essential to early diagnose asymptomatic patients with COVID-19. CT scan has great sensitivity in screening and detecting patients with COVID-19 pneumonia.

## Background

COVID-19, formerly known as 2019 novel coronavirus (2019-nCoV), was declared to be a global health emergency by the World Health Organization on 30th January 2020 [1].

CT imaging patterns and radiological examinations have become vital in early diagnosis and assessment of disease course [2].

Thin-slice chest CT plays a vital role in early detection, observation, and disease evaluation [2].

Radiological examinations are vital in early diagnosis and assessment of disease course, as most COVID-19 infected patients were diagnosed with pneumonia and characteristic CT imaging patterns [3].

Asymptomatic infected individuals, called “asymptomatic carrier or transmitter”, may also become the contagious source of SARS-CoV-2, and some of them progress rapidly, even resulting in acute respiratory distress syndrome (ARDS) with a high case-fatality rate [4, 5].

In the absence of specific therapeutic drugs or vaccines for 2019 novel coronavirus disease (COVID-19), it is essential to detect the diseases at an early stage and immediately isolate the infected person from the healthy population [6].

The low sensitivity of RT-PCR implies that many COVID-19 patients may not be identified and may not receive appropriate treatment in time; such patients constitute a risk for infecting a larger population given the highly contagious nature of the virus [7].

Chest CT, as a routine imaging tool for pneumonia diagnosis, is relatively easy to perform and can produce fast diagnosis [8].

Chest CT is a conventional, non-invasive imaging modality with high accuracy and speed. Based on available data published in recent literature, almost all patients with COVID-19 had characteristic CT features in the disease process [6].

The chest CT scans showed a higher sensitivity for the diagnosis of COVID-19 infection than initial RT-PCR results [9].

Imaging is a critical component of the diagnostic workup, monitoring of disease progression, and follow-up in coronavirus-related pulmonary affection [10].

The aims of our study are to understand the imaging spectrum of asymptomatic COVID-19 positive patients and to facilitate the detection and isolation of asymptomatic patients who act as the asymptomatic transmitter of the disease.

## Methods

### Patients

This cross-sectional study included 44 asymptomatic patients (16 males, 28 females) with an age range from 8 to 66 years (mean age of 35.7 years), who were not yet diagnosed as asymptomatic corona patients; then, after imaging, they were confirmed to be infected with SARS-CoV-2 using RT-PCR test, they were referred for MSCT assessment of the chest for different causes (Table 1). MSCT of the chest was done to all patients as requested. The study was conducted between March 20 and May 20, 2020 in Cairo, Egypt.

**Table 1:** Clinical history of patients enrolled in the study

Number of patients	Medical history
31 (70.45%)	Positive recent contact with proved COVID-19 cases
9 (20.45%)	Recent travel history
2 (4.54%)	Pre-hospital admission for different surgeries
2 (4.54%)	For routine checkup in non-metastatic cancer patients

#### Inclusion criteria

Laboratory proven PCR positive COVID-19 tests

Asymptomatic patient (regarding chest symptoms)

#### Exclusion criteria

Patients who experienced clinically defined pulmonary infection symptoms Pregnant females

Patients with severe artifacts on CT images

Patients with chest symptoms

### Methods

All symptomatic patients were subjected to:

- ❖ Through history taking
- ❖ MSCT of the chest was done to all patients

- Using a multi-detector CT scanner with 32 or more channels
- The detailed parameters for CT acquisition were as follows:
- Tube voltage, 120–160 kVp
- Tube current, standard (reference mAs, 60–120)
- Slice thickness, 1.0 mm
- Reconstruction interval, 1.0–3.0 mm
- Using a sharp reconstruction algorithm
- CT images were obtained with the patient in the supine position with suspended full inspiration and without contrast medium
- Then, acquired images were sent to a separate workstation to be processed, manipulated and reconstructed in axial, coronal, and sagittal planes to detect the craniocaudal and axial distribution of parenchymal affection (2D Multiplanar Images Reconstruction) (MPR)
- All images were viewed on both lung (width, 1500 HU; level, −700 HU) and mediastinal (width, 350 HU; level, 40 HU) settings.
- The chest CT scan was evaluated by two expert radiologist separately searching for the following characteristics:
- Presence of ground-glass opacities with their axial and centrilobular distribution.
- Presence of consolidation
- Laterality of ground-glass opacities and consolidation
- Presence of nodules
- Presence of a pleural effusion
- Airways abnormalities (including airway wall thickening, bronchiectasis
- Other abnormalities, including linear opacities, opacities with rounded morphology, opacities with “halo and reverse halo” sign and opacities with a “crazy-paving” pattern.

#### Statistical analysis

Owing to small sample size, findings are presented as medians and interquartile ranges

## Results

This cross-sectional study included 44 asymptomatic patients (16 males, 28 females) with age ranging from 8 to 66 years (mean age of 35.7 years), after imaging, they were confirmed to be infected with SARS-CoV-2 using RT-PCR test, they were referred for MSCT assessment of the chest for different causes.

Most patients presented with a past history of close contact with COVID-19 positive patients (31, 70.45%), while others gave recent travel history (9, 20.45%), two patient came for preoperative assessment (2, 4.45%) and two patients came for their routine follow up (2, 4.45%) (Table 1).

The most prominent radiological feature was ground-glass opacity. Including simple ground glass found in 28 patients (63.6 % of cases), ground glass with thickened interlobular setae in 8 cases (18.8%), ground glass with curvilinear subpleural line (1, 2.27%) and ground glass with halo sign (4, 9.09%). Another CT feature was consolidation found in 3 patients (6.81 %) of cases (Table 2).

**Table 2:** showing various radiological features

Radiological feature	Number of patients	Percent of patients
Ground glass (total)	41	93%
Simple ground glass	28	63.6%
Ground glass + reticulation	8	18.18%
Ground glass with halo sign	4	9.09%
Ground glass with subpleural curvilinear line	1	2.27%
Consolidation	3	6.81%

From the 41 cases presented with ground-glass opacification, 28 patients (63.6 %) where simple ground glass, 8 patients (18.8 %) ground glass was associated with fine reticulation.

The ground-glass and consolidative opacities were peripheral in most patients (34, 77.3%), while 3 patients(6.81%) showed peri-hilar distribution and 7 patients (15.9%) showed peripheral with perihilar involvement (Table 3).

**Table 3:** Showing axial and craniocaudal predominant distribution

Predominant distribution	Number of cases	Percent
Bilateral	20	45.5%
Peripheral only	34	77.3%
Peri-hilar only	3	6.81%
Peripheral and peri-hilar	7	15.9%
Right upper lobar	14	31.8%
Right lower lobar	22	50%
Right middle lobe	2	4.5%
Left upper lobe	14	31.8%
Left lower lobe	32	72.7%

Twenty patients (20, 45.5%) showed bilateral affection.

Thirty-two patients showed left lower lobe predominance (72.7%), followed by the right lower lobe predominance in 22 patients (50%) (Table 3).

Reversed halo sign, pleural effusions, pericardial effusion, cavitation, mediastinal, and hilar lymph node enlargement were not seen in any of the patients.

## Discussion

Coronavirus disease 2019 (COVID-19) is a highly infectious disease caused by severe acute respiratory syndrome coronavirus 2 (SARS-CoV-2) [11].

As of 21 May 2020, the number of cases of confirmed COVID-19 globally is over 5 million [12].

CT examination is of great significance not only in diagnosing COVID-19 but also in monitoring disease progression and evaluating therapeutic efficiency [13].

Asymptomatic infected individuals, called “asymptomatic carrier or transmitter”, may also become the contagious source of SARS-CoV-2, and some of them progress rapidly, even resulting in acute respiratory distress syndrome (ARDS) with a high case-fatality rate [4, 5].

This cross-sectional study included 44 asymptomatic patients confirmed to be infected with COVID-19 by PCR study. MSCT of the chest was done to all patients as requested. The study was conducted between March 20 and May 20, 2020, in Cairo, Egypt.

This study included 16 males and 28 females with an age range from 8 to 66 years.

The mean age of the studied patients was 35.7 years with female predominance 63.6 %.

All the patients were asymptomatic. Most of them gave a history of close contact to COVID positive patients (70.45 % of cases), recent travel history (20.45 % of cases), while 2 patients came for preoperative assessment (4.45%) and 2 came for an annual checkup (4.45%).

MSCT chest showed abnormalities in all patients.

**Fig. 1:**
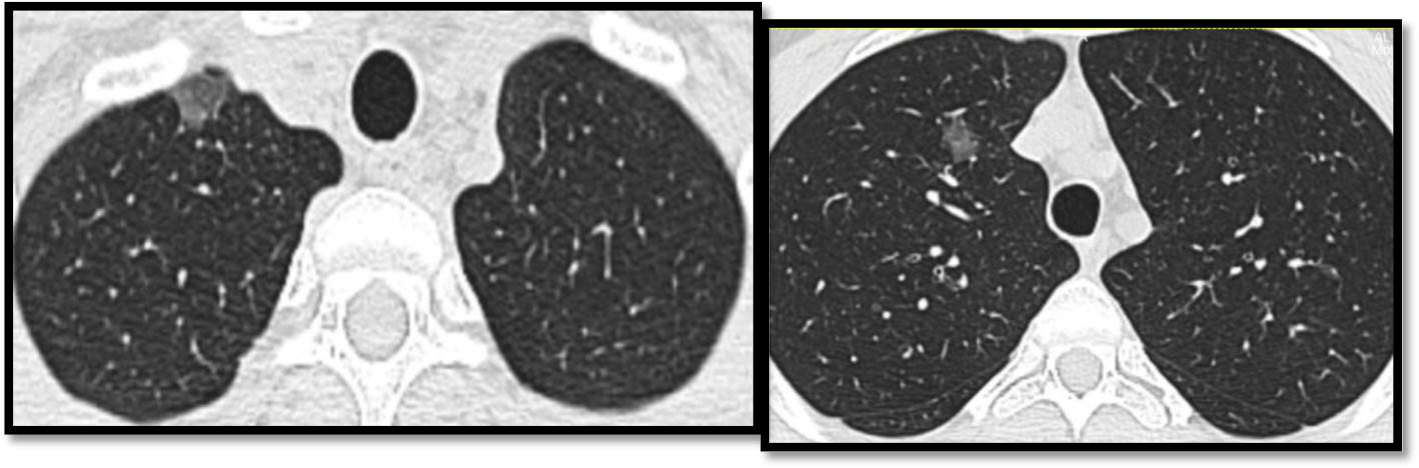
An 18-year-old male patient with no chest symptoms gave a history of close contact to positive COVID-19 patient. MDCT showed small patchy ground-glass opacities GGO seen at the right upper lung lobe

In our study, we noted that ground-glass opacity was the predominant radiological finding (41, 93%) which agrees with the study done by Heng et al. who noted that ground glass was the most evident radiological finding in COVID-19 positive asymptomatic patients [14] (Fig. 1).

**Fig. 2:**
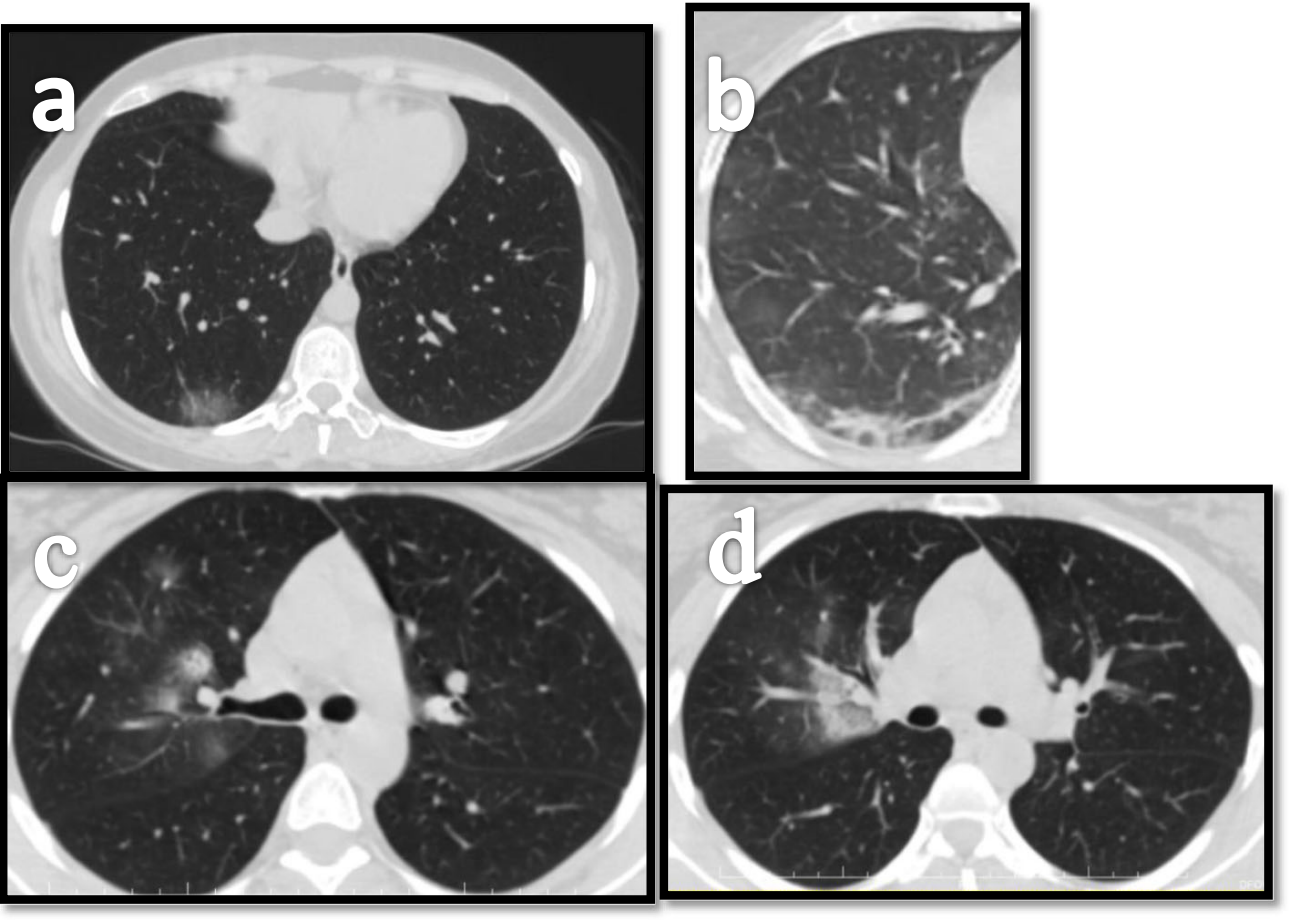
A group of asymptomatic patients showing different radiological chest CT findings denoting early COVID. **a** Single subpleural basal pure GGO. **b** Subpleural curvilinear atelectasis. **c** Nodular consolidation with surrounding ground-glass veiling giving halo sign. **d** Fissural-based ground-glass opacity showing coarse interstitium within

We have noticed simple GGO in 28 cases (63.6%), GGO with interlobular septal thickening in 8 cases (18.8 %), GGO with halo sign in 4 cases (9%), and GGO with subpleural curvilinear line in 1 case (2.27%), compared with the results of Heng et al., who found that simple GGO in 51.7%, GGO with fine reticulation in 12.1%, GGO with halo sign in 8.6%, and GGO with subpleural curvilinear line in 10.3% [14] (Fig. 2).

**Fig. 3:**
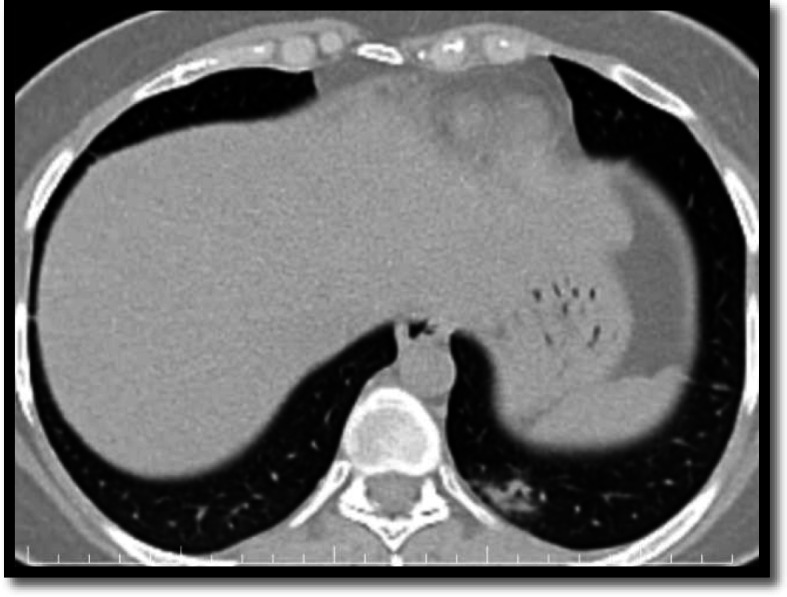
A 49-year-old female patient with no chest manifestations. MDCT showed left lower lung lobe basal segment small consolidation with air bronchogram seen within

Consolidation was seen in 3 patients (6.81 %) of cases, which agrees to the results of Heng’s study who found consolidation in 5.2% of patients (Fig. 3).

The ground-glass and consolidative opacities were peripheral in most patients (34, 77.3%), while 3 patients(6.81%) showed peri-hilar distribution and 7 patients (15.9%) showed peripheral with perihilar involvement which is highly matching results of the study” CT imaging and clinical course of asymptomatic cases with COVID-19 pneumonia” done by Ming et al. which found that the lesions mostly located in peripheral (44, 75.9%), and 14 (24.1%) patients presented central distribution [14].

**Fig. 4:**
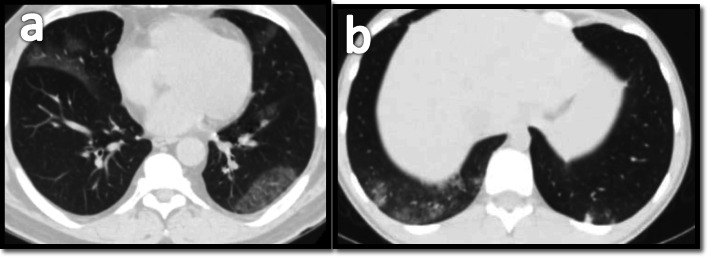
MSCT scan for two asymptomatic patients. **a** 33-year-old male patient with recent travel history showing bilateral variable-sized patchy GGO seen affecting middle lobe, left lower lobe, and lingua being peripheral sub-pleural in location. **b** 41-year-old female patient giving a history of close contact to +ve PCR COVID-19 patient showing bilateral lower lung lobes small GG nodular opacities predominantly at the peripheral sub-pleural location

Less than half of the patients (20, 45.5%) presented bilateral lesions; 24 (54.5%) patients showed unilateral lung distribution. That was not matching with Heng’s study who found that unilateral involvement was more common (59%). While our results matching a study done by Allan who found that abnormalities were bilateral in 86% of cases, mostly evident in the lower lobes and peripheral lung zones [14, 15] (Fig. 4).

**Fig. 5:**
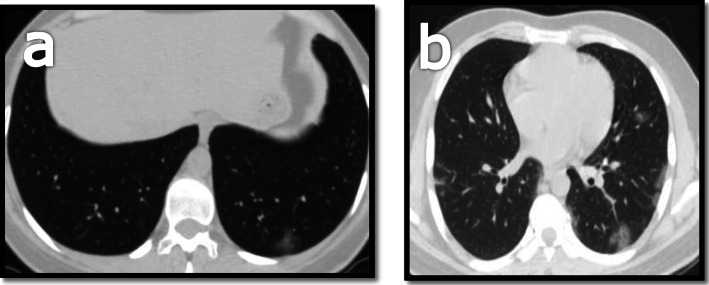
**a** MDCT scan for a 28-year-old male patient showing left lower lung lobe basal subpleural simple GGO. **b** MDCT scan for a 39-year-old male patient showing bilateral mainly peripheral subpleural GGO more predominant at the left lower lung lobe

The left lower lobe showed the highest predominance (32, 72.7%), followed by the right lower lobe (22, 50%) and equal incidence in both upper lobes (31.8 %), which is matching result of Allen’s and Heng’s study who showed lower lobe predilection of the disease [14, 15] (Fig. 5).

Reversed halo sign, pleural effusions, pericardial effusion, cavitation, mediastinal, and hilar lymph node enlargement were not seen in any of our patients

## Conclusion

CT images of asymptomatic cases with COVID-19 pneumonia have definite characteristics.

As the COVID-19 pandemic continues to claim lives across the globe, early diagnosis of asymptomatic COVID-19 patients is essential as they act as a covert transmitter. Once diagnosed, limiting their physical contact with others is one way to slow the spread.

Although the use of reverse transcriptase-polymerase chain reaction (RT-PCR) is the gold standard, yet, RT-PCR is not 100% accurate, as there are false-positive and false-negative test results.

In China, where experts quickly and effectively controlled the disease, professional medical organizations universally agree that CT plays “a vital role in early detection, observation, and disease evaluation.”

So, our study emphases the rule of CT chest in early diagnosis of asymptomatic COVID patients and thus reduce the spread of such epidemic.

## Data Availability

Data available within the article or its supplementary materials.

## Abbreviations

2019-nCOV: 2019 novel coronavirus
ARDS: Acute respiratory distress syndrome
COVID-19: Coronavirus disease
CT: Computed tomography
GGO: Ground-glass opacity
IV: Intravenous
MDCT: Multi-detector computed tomography
MSCT: Multi-slice computed tomography
RT-PCR: Reverse transcriptase-polymerase chain reaction
PCR: Polymerase chain reaction
SARS-Cov-2: Severe acute respiratory syndrome coronavirus 2
